# Cardiopulmonary Sleep Spectrograms Open a Novel Window Into Sleep Biology—Implications for Health and Disease

**DOI:** 10.3389/fnins.2021.755464

**Published:** 2021-11-12

**Authors:** Haitham S. Al Ashry, Yuenan Ni, Robert J. Thomas

**Affiliations:** ^1^Division of Pulmonary and Sleep Medicine, Elliot Health System, Manchester, NH, United States; ^2^Division of Pulmonary, Critical Care and Sleep Medicine, West China Hospital, Sichuan University, Chengdu, China; ^3^Division of Pulmonary and Sleep Medicine, Beth Israel Deaconess Medical Center, Boston, MA, United States

**Keywords:** cardiopulmonary coupling (CPC), heart rate variability, sleep apnea, stable sleep, insomnia

## Abstract

The interactions of heart rate variability and respiratory rate and tidal volume fluctuations provide key information about normal and abnormal sleep. A set of metrics can be computed by analysis of coupling and coherence of these signals, cardiopulmonary coupling (CPC). There are several forms of CPC, which may provide information about normal sleep physiology, and pathological sleep states ranging from insomnia to sleep apnea and hypertension. As CPC may be computed from reduced or limited signals such as the electrocardiogram or photoplethysmogram (PPG) vs. full polysomnography, wide application including in wearable and non-contact devices is possible. When computed from PPG, which may be acquired from oximetry alone, an automated apnea hypopnea index derived from CPC-oximetry can be calculated. Sleep profiling using CPC demonstrates the impact of stable and unstable sleep on insomnia (exaggerated variability), hypertension (unstable sleep as risk factor), improved glucose handling (associated with stable sleep), drug effects (benzodiazepines increase sleep stability), sleep apnea phenotypes (obstructive vs. central sleep apnea), sleep fragmentations due to psychiatric disorders (increased unstable sleep in depression).

## Introduction

The prevalence of sleep disorders has been increasing over the last two decades (Acquavella et al., 2020). Disorders like insomnia and sleep apnea have a prevalence of as much as 20% in the general population (Franklin and Lindberg, 2015; Acquavella et al., 2020). There is a need for nimble sleep state estimation, diagnostics, and tracking. One approach seeing increasing utilization both in formal medical and consumer wearable devices is through analysis of heart rate and respiration. There is a strong correlation between changes in heart rate variability and sleep during health and disease (Tobaldini et al., 2013). High frequency (HF) components mainly present parasympathetic activity, while low frequency (LF) components is partly a quantitative marker of sympathetic modulation. LF and the LF/HF ratio are high in Wake and decrease in NREM sleep, peaking once more during REM sleep, while HF follows the opposite trend. Deep NREM sleep (N3) typically has the greatest HF power. Sleep disruptive influences such as sleep apnea (Qin et al., 2021; Ucak et al., 2021), insomnia (Spiegelhalder et al., 2011; Dodds et al., 2017; Cosgrave et al., 2021), and depression (Hyunbin et al., 2017; Gao et al., 2019; Eddie et al., 2020) are associated with an increase in the LF components.

Different techniques have been used to assess for such changes one of which is analysis of cardiopulmonary coupling and coherence (CPC) patterns. In this technique a single lead electrocardiogram (ECG) or photo plethysmogram (PPG) is used to extract heart rate variability and ECG or PPG signal derived respiration (EDR/PDR) (Thomas et al., 2005; Hilmisson et al., 2020). Contrary to the stage/grade approach to conventional sleep characterization, CPC analysis based on coupling and coherence provides a novel and complementary view of sleep, that of bistability, which are particularly well defined during NREM sleep. Thus, distinct patterns of CPC are observed: high frequency coupling (HFC) which is associated with stable NREM sleep and low frequency coupling (LFC) which is associated with unstable and often fragmented NREM sleep (Thomas et al., 2005). A third CPC pattern named very low frequency coupling occur in both REM sleep and wake, which may be differentiated by analysis of signal quality and motion artifact (Al Ashry et al., 2021). High and low frequency coupling are mutually exclusive and shift logically with disease states and treatments. For example, there is an increase LFC in patients with insomnia and this can be tracked in the ambulatory setting (Thomas et al., 2017a,b). There is increased LFC during sleep in unmedicated patients with major depression and improvement with therapy of major depression (Yang et al., 2011). Integrating CPC with oximetry allows generating a true FDA approved apnea-hypopnea index (AHI), which shows good correlation with conventional polysomnogram-derived AHI (Hilmisson et al., 2020; Al Ashry et al., 2021).

The overall goal of this article is to review physiological basis, techniques, and applications of CPC spectrograms in sleep in health and disease. There are three aims for this review, to show that—(a) CPC shows a fundamental characteristic of NREM sleep—bimodality, across a number of physiologies; (b) CPC has several uses in sleep apnea care—diagnosis, phenotype, tracking outcomes; (c) CPC can diagnose and track non-apneic sleep fragmentation and medication effects, and should be used in the appropriate clinical context.

## Physiology Background

Entrainment between heart rate and respiration in humans has been described since the early twentieth century (Galletly and Larsen, 1998). It has been suggested that such synchrony between heart rate and respiration improves pulmonary gas exchange and computational models have shown that healthy cardiopulmonary coupling minimizes the heart workload while maintaining adequate ventilation (Yasuma and Hayano, 2004; Ben-Tal et al., 2012). Such strong cardiopulmonary coupling is seen at its best during deep sleep, sedation, and anesthesia (Dick et al., 2014). There is a critical influence of the autonomic nervous system on cardiopulmonary coupling (Bartsch et al., 2012). Non-rapid eye movement (NREM) sleep is associated with decreased sympathetic activity, a decrease in heart rate, and a decrease of average blood pressure and blood pressure variability in comparison to the wake state (Somers et al., 1993). Respiratory sinus arrythmia is a phenomenon in which the heart rate variability is synchronized beat-to-beat with respiration and is most pronounced during deep NREM sleep (Zemaityte et al., 1984; Yasuma and Hayano, 2004). In contrast, during rapid eye movement (REM) sleep there is dominance of sympathetic control and a burst frequency of sympathetic activity that is actually higher than during wakefulness leading to increases in blood pressure variability and heart rates similar to what is seen during wakefulness (Somers et al., 1993). Using spectral analysis, a frequency of 0.1 Hz and above has been associated with parasympathetic activity dominance and frequencies below 0.1 Hz have been associated with dominance of sympathetic activity (Appel et al., 1989; Cui et al., 2020).

## Cardiopulmonary Coupling Methodology

Cardiopulmonary sleep spectrograms were first obtained from a single lead ECG (Thomas et al., 2005, 2007). ECG-derived respiration (EDR) is obtained either by using R-S wave amplitudes or variations in QRS complexes area (Zheng et al., 2016). Several studies have looked at improving the accuracy of deducing EDR from single lead ECG and reducing noise but is beyond the scope of this paper (Thayer et al., 1996; Leanderson et al., 2003; Liu et al., 2012; Zheng et al., 2016). In parallel to extracting the EDR, ectopic beats are identified and removed and normal sinus—normal sinus (NN) intervals are extracted and outliers are filtered (Thomas et al., 2005). After extracting the N-N interval series on ECG and its associated EDR, the signals are then resampled using cubic splines at 2 Hz. The Fast Fourier Transform is applied to 3 overlapping 512 sample sub-windows within the 1,024 coherence window. The 1,024 coherence window is then advanced by 256 samples (2.1 min) and the calculation repeated until the entire N-N interval/EDR series is analyzed. Thus, the cross-spectral power and coherence of these two signals are calculated over a 1,024 sample (8.5 min) window.

**FIGURE 1:**
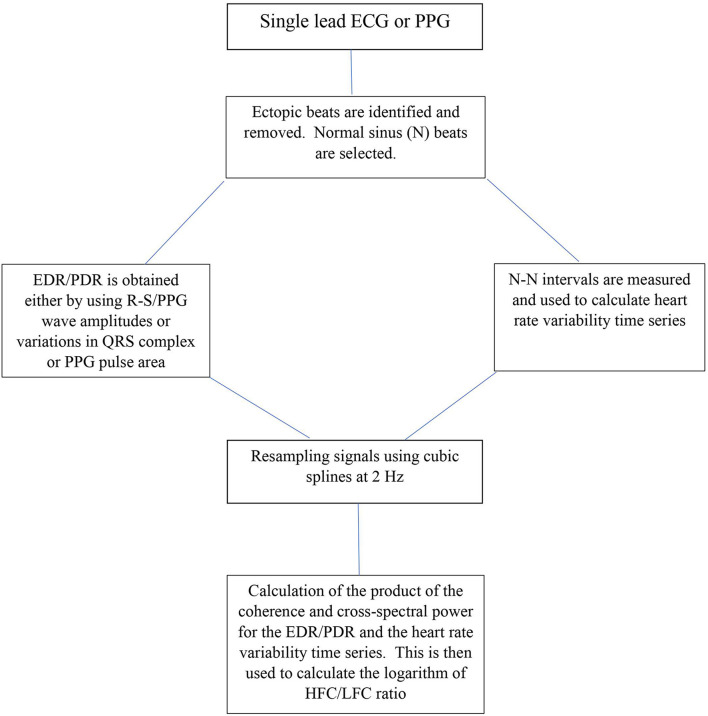
Schematic for CPC analysis. ECG, Electrocardiogram; PPG, Photoplethysmogram; EDR, ECG-derived respiration; PDR, Photoplethysmogram derived respiration R-S and QRS are ECG waveforms; N-N intervals, Normal sinus to normal sinus intervals; Hz, frequency; HFC, high frequency coupling; LFC, low frequency coupling.

For each 1,024 window the product of the coherence and cross-spectral power is used to calculate the ratio of coherent cross power in the low frequency (0.01–0.1 Hz.) band to that in the high frequency (0.1–0.4 Hz.) band. The logarithm of the high to low frequency cardiopulmonary coupling ratio [log (HFC/LFC)] is then computed to yield a continuously varying measure of cardiopulmonary coupling (Al Ashry et al., 2021). While originally the ECG signal was used as input, any signal or signal set which encodes respiration and heart rate variability may be used to compute the CPC sleep spectrogram. Figure 1 shows the steps in computing CPC.

## Distinct Cardiopulmonary Coupling Patterns in Sleep

### Sleep Stages and Cyclic Alternating Pattern

The conventional characterization of sleep stages dictates a “graded” approach to NREM sleep, from lightest (N1) to deepest (N3). The difference between N2 and N3 are relatively arbitrary, dependent on the proportion of high amplitude slow waves (20% threshold) for a given epoch. However, there is a great variability of depth of NREM sleep, as can be readily objectively demonstrated by techniques such as the Odds Ratio Product (Younes et al., 2015). A unique and key feature of CPC sleep states is poor correlation of HFC and LFC with conventional NREM sleep stages. Thus, in health, the majority if N2 is also HFC, N3 is usually HFC but at times LFC, while N1 is always LFC. There is a moderate correlation with a well-described stability dimension of NREM sleep, Cyclic Alternating Pattern (CAP) (Thomas et al., 2005). CAP is a distinct pattern that can be seen on electroencephalography (EEG) during unstable NREM sleep. High frequency coupling dominates when CAP is sparse or absent, while LFC is reliably associated with CAP. Conventional NREM stage N3 is usually HFC, but so is the majority of healthy N2, where non-CAP periods also dominate. Thus, CAP and CPC capture significantly overlapping domains of NREM sleep stability while both measures correlate only partially with conventional measures of sleep depth.

### Slow Wave (Delta) Power

Slow-wave power in the sleep EEG has highly characteristic spatial and temporal evolution patterns across a night. Power in the 1–4 Hz frequencies dominates the first half of the night, but the ebb and flow of slow-wave power continues throughout the night. High frequency coupling strongly covaries with slow-wave power across the whole night, while low frequency power in heart rate variability is inversely related to EEG delta power (Brandenberger et al., 2001; Ako et al., 2003; Thomas et al., 2014). One interesting finding when aligning HFC with delta power is that there is a consistent lag of delta power after HFC where HFC usually precedes an increase in delta power by an average of 6 min, suggesting that subcortical/brain-stem mechanisms may lead large-scale cortical synchrony during sleep (Thomas et al., 2014).

### Sleep Blood Pressure

A key dimension of health is a reduction of blood pressure during sleep (blood pressure “dipping”). Sleep blood pressure is known to dip during stage N3, and rise during REM sleep, yet the majority of sleep is N2, and dipping profiles occupy the entire sleep period, not just N3-enriched zones. This discrepancy has been solved by correlating blood pressure during sleep with stable sleep as determined by CPC (high frequency coupling). Blood pressure dipping occurs only during HFC periods (Wood et al., 2020). In a randomized trial targeting sleep apnea treatment in patients with cardiovascular risk factors, it was shown that those who were treated with CPAP had more HFC during sleep, which was in turn associated with improvement in blood pressure dipping and mean arterial pressures during sleep (Magnusdottir and Hilmisson, 2018).

### Autonomic Regulation During Sleep

**FIGURE 2:**
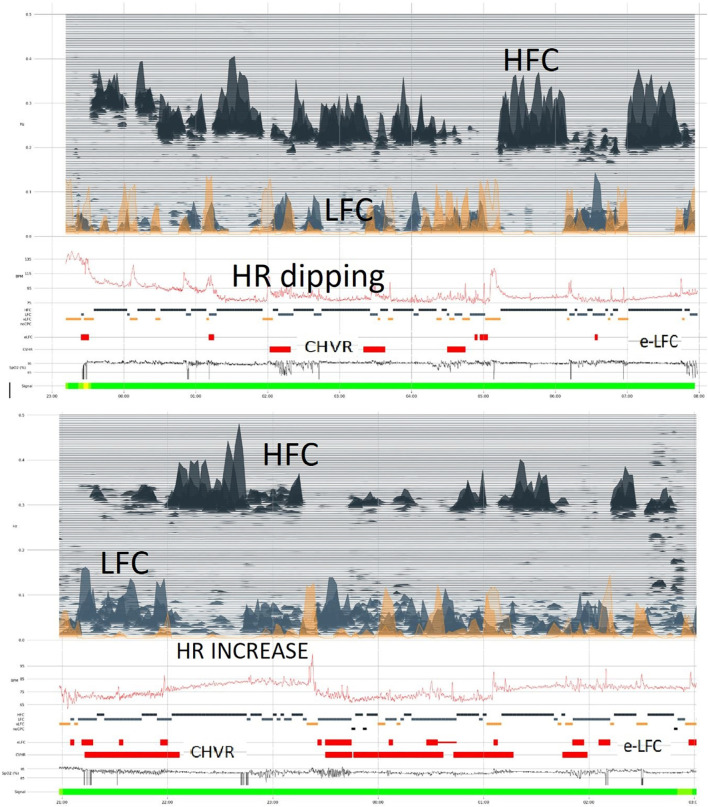
Photoplethysmogram/oximetry-based CPC-heart rate analysis. The oximetry-based analysis provides a full CPC-sleep spectrogram, an apnea-hypopnea index by integrating CPC LFC and oxygen desaturation events, and a profile of heart rate across the night. In the upper segment of the figure, “dipping” of heart rate is noted along with abundant high frequency coupling/stable sleep. In the lower sample, there is less stable state, but the heart rate profile is distinctly abnormal, with an elevation even during stable state. Such relative tachycardia during stable NREM sleep may suggest obstructive hypoventilation. In both examples, oxygen desaturation itself is mild. Stable and unstable sleep (HFC and LFC, respectively) occur intermittently through the night. HFC, high frequency coupling; LFC, low frequency coupling; e-LFC, elevated low frequency coupling; HR, heart rate; CHVR, cyclic heart rate variation.

There is normally a reduction of the heart rate (HR) during sleep, a HR-dip, which roughly follows the blood pressure dipping pattern. Heart rate during sleep is, however, far simpler to measure than blood pressure and provides a window into cardiovascular health. By aligning heart rate profiles with the CPC spectrogram (Figure 2), unique insights and cardiovascular risk profiles are potentially extractable, and can be tracked over time. Normally, HR dipping occurs during HFC periods.

### Vertically Integrated Multi-Component Sleep States

**FIGURE 3:**
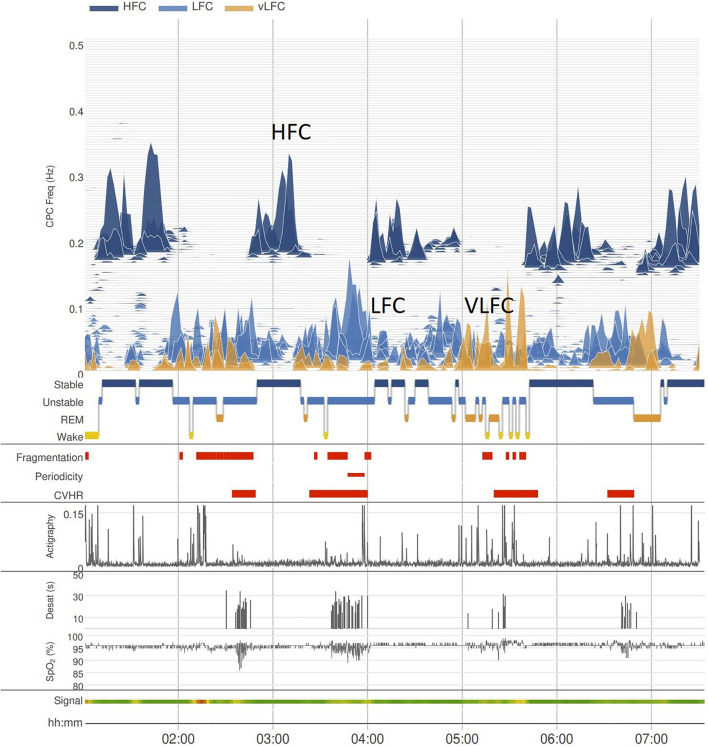
The oximeter-extracted CPC spectrogram. The basic graphical representation of the CPC-spectrogram has high, low, and very low frequency coupling (HFC, LFC, and VLFC, respectively) components. Actigraphy is integrated, and VLFC without movement is considered REM sleep, whereas VLFC with movement is Wake. Cyclic variation of heart rate is also displayed, as well as e-LFC as a measure of sleep fragmentation. The oximeter signal itself provides standard oximetry metrics, such as an oxygen desaturation index. As shows, periods of HFC and LFC alternate throughout the night. LFC, low frequency coupling; VLFC, very low frequency coupling; HFC, high frequency coupling; CHVR, cyclic heart rate variation.

Cardiopulmonary coupling analysis established that sleep is bimodal than graded. That is, while conventional NREM sleep stages moves across the N1 to N3 grades, the CPC-spectrogram shows that NREM sleep has only two distinct and completely non-overlapping forms—stable and unstable (HFC and LFC, respectively), which intermittently switch across the entire night. While N3 dominates in the first half of the night, HFC occurs throughout (Figure 3). This bimodality or stability domain is especially clear when incorporating autonomic and respiratory variables with electrocortical activity, specifically, delta power and the < 1 Hz slow oscillation. Stable NREM is characterized by high probability of occurrence of the < 1 Hz slow oscillation, high delta power, non-CAP EEG, stable breathing, blood pressure dipping, strong sinus arrhythmia and vagal dominance, and high frequency CPC. Conversely, unstable NREM exhibits opposite features: a fragmented and discontinuous < 1 Hz slow oscillation, CAP patterns on the EEG, non-dipping of blood pressure, unstable respiration, cyclic variation in heart rate, and low frequency CPC (Wood et al., 2020).

## Cardiopulmonary Coupling in Sleep Apnea

### Diagnosis of Sleep Apnea

Sleep apnea reliably induces strongly coupled low-frequency oscillations in heart rate and respiration. This results in strong ECG or PPG amplitude fluctuations, besides cyclic variation in heart rate, enabling computing an AHI. This computation requires knowing the number of oxygen desaturation events, the amount of time in coupled low-frequency oscillations, the mean frequency of these computed oscillations, and the total sleep period.

The first step in using CPC for sleep apnea detection involves a second-levels analysis of the LFC zone, where a spectral band designated as elevated-LFC (e-LFC) was found which correlated highly with scored apneas and hypopneas. Within e-LFC, two further patterns were discernable, one with a wide dispersion of coupling spectra and another with a narrow band of coupling spectra (broad and narrow-band e-LFC, or e-LFC_BB_ and e-LFC_NB_). However, other causes of sleep fragmentation may also cause similar patterns, especially e-LFC_BB_, a limitation which may be minimized by integrating oxygen saturation fluctuations into the computation. In two recent large studies combining CPC AHI with oximetry desaturation index events in one index have improved the accuracy of derived AHI in comparison to PSG AHI (Hilmisson et al., 2020; Al Ashry et al., 2021). This derived AHI was approved to be equivalent to PSG AHI in adults and children in 2019 by the FDA (K182618).

These LFC_NB_ and LFC_BB_ indices have been used in several studies in the adult and pediatric populations for automated detection of sleep apnea (Table 1, section A). There are several advantages for using CPC through wearable devices, especially the current embodiment of a ring-form oximeter, in the sleep apnea population. These include: (1) cost-effective screening of high risk adult and pediatric populations; (2) minimizing patient (wearing) and system (scoring) burdens; (3) detection of expressed high loop gain (central apnea and periodic breathing), which can be a risk stratification approach, as such patients are at risk for treatment-emergent central sleep apnea, reduced adherence to therapy, and persistent respiratory instability during apnea therapy.

**TABLE 1:** Studies that used cardiopulmonary coupling to diagnose and follow treatment response in various sleep disorders.

**Study year**	**Number of subjects**	**Results**
**Section A: Studies that used cardiopulmonary coupling analysis to generate automated apnea-hypopnea index**		
Roche et al. (2003)	147	Sensitivity of 92.4% and a specificity of 90.1%
Roche et al. (2004)	63	Receiver operating characteristic (ROC) showed area under the curve of 0.848
Vazir et al. (2006)	33	Sensitivity of 85%, specificity of 65%, positive predictive value of 61%, and a negative predictive value of 87%.
Heneghan et al. (2008)	92	Correlation coefficient between the CPC AHI and PSG AHI is: (*r* = 0.88, *p* < 0.001)
Chang (2009)	60	sensitivity of 68.97% and specificity of 100%
Al-Abed et al. (2009)	14	sensitivity, specificity and accuracy were 100.0, 99.9, and 99.9%, respectively
Hayano et al. (2011)	862	Receiver operating characteristic (ROC) showed area under the curve (AUC) of 0.913 for subjects with AHI 15 or higher
Guo et al. (2011)	63 children (mean age 6.2 years; range 2–12 years)	Compared CPC AHI to AHI from portable sleep testing device and found correlation coefficient of 0.70
Liu et al. (2012)	69	Receiver operating characteristic (ROC) showed area under the curve of 0.79 in detecting apneas and hypopneas
Magnusdottir and Hilmisson (2018)	47 subjects with moderate to severe OSA with AHI 15 or higher	Sensitivity 89%, specificity 79%, agreement 85%, PPV (positive predictive value) 0.86, and NPV (negative predictive value) 0.83
Hilmisson et al. (2019a)	42 subjects with moderate to severe OSA with AHI 15 or higher	Sensitivity of 100%, specificity of 81%, and agreement of 93%
Lu et al. (2019)	179	ROC showed AUC 0.79 in mild OSA, 0.79 in moderate OSA, and 0.86 in severe OSA
Hilmisson et al. (2020)	805 children with mean age of 6.8 years	ROC demonstrated strong agreement in all OSA categories: 91.4% in mild OSA; 96.7% in moderate OSA; 98.6% in severe OSA
Ma et al. (2020)	205	Correlation coefficient between the CPC AHI and PSG AHI is: (*r* = 0.851, *p* < 0.001)
Seo et al. (2020)	194	Correlation coefficient between the CPC AHI and PSG AHI is: (*r* = 0.973, *p* < 0.001)
Al Ashry et al. (2021)	833	ROC demonstrated strong agreement in all OSA categories: 98.5% in mild OSA; 96.4% in moderate OSA; 98.5% in severe OSA

**Study year**	**Number of subjects**	**Results**

**Section B: Studies that used cardiopulmonary coupling in phenotyping and following response of treatment of sleep apnea**		
Roche et al. (1999)	Cohort of 14 patients with OSA treated with CPAP for 3 months	Follow up PSGs showed that AHI decreased from average of 50 to 2/h and this was associated with significant reduction in the LFC/HFC ratio
Gilman et al. (2008)	Randomized control study in 19 patients with heart failure	The group treated with CPAP for 1 month had significant increase in HFC compared to the group not treated with CPAP
Shiina et al. (2010)	Cohort of 50 patients with AHI > 20/h tested before and after 3 months of CPAP therapy	There was significant decrease in LFC/HFC ratio and C-reactive protein after 3 months of CPAP
Schramm and Thomas (2012)	Case report of patient with mild OSA. Multiple ECG recording nights obtained. No therapy, dental device, oxygen therapy, and positional therapy were compared	HFC/LFC ratio significantly improved on the night of dental device as compared to oxygen therapy and positional therapy.
Lee et al. (2012)	Cohort of 37 children with OSA after adenotonsillectomy	AHI determined by PSG decreased significantly after adenotonsillectomy. This was associated with significant improvement in HFC/LFC ratio
Harrington et al. (2013)	Cohort of 24 patients undergoing CPAP titration PSG for OSA. A successful titration was defined as AHI < 5/h.	HFC was decreased and LFC was increased in subjects with unsuccessful CPAP titrations.
Ramar et al. (2013)	Cohort of 106 patients with complex sleep apnea undergoing ASV titration	No correlation was found between percentage of LFC_*NB*_ and ASV titration success
Lee et al. (2014)	Cohort of 52 patients with OSA treated with dental devices. PSG and CPC were obtained at baseline and 3 months into treatment	The reduction in AHI as assessed by PSG was associated with increase in HFC and decrease in LFC
Choi et al. (2015)	Cohort of 62 patients with OSA treated with surgery.	36 patients had a successful surgical outcome defined as 50% reduction of AHI to AHI < 20/h and were found to have significant increase in HFC and significant decrease in LFC compared to those who didn’t have a successful surgical outcome
Lee et al. (2016)	Cohort of 98 patients with OSA treated with surgery or dental device. PSG and CPC were obtained at baseline and 3 months into treatment	Patients who had > 50% reduction in their AHI 3 months after treatment were found to have significant reduction in LFC and significant increase in HFC
Cho and Kim (2017)	Cohort of 62 patients tested in the sleep lab for OSA	In those who met criteria for split night the CPAP titration portion was associated with significant increase in HFC and significant decrease in LFC
Chen and He (2019)	Case control study in a pediatric population with OSA. The control group underwent adenoidectomy only whereas the intervention group underwent drug-induced sleep endoscopy (DICE) and tonsillectomy was performed in addition to adenoidectomy if tonsillar obstruction was seen on DICE	Both groups had improvement in AHI as determined by CPC but AHI improvement in the DICE group after 1 year was better as compared to the control group.

**Study year**	**Number of subjects**	**Results**

**Section C: Studies that used cardiopulmonary coupling in sleep disorders other than sleep apnea**		
Sforza et al. (2005)	Cohort of 14 patients with periodic limb movements (PLMs) was studied. Periods of PLMs were compared to periods without PLMs	Periods with PLMs were associated with significant increase in LFC compared to periods without PLMs
Thomas et al. (2010)	Prospective case control study. 14 patients with fibromyalgia were compared to 13 matched controls	Elevated-low frequency coupling was significantly increased in Fibromyalgia patients and there was a trend toward less HFC in those patients as compared to controls
Yang et al. (2011)	100 patients with major depression (50 of which were on hypnotics due to insomnia) were compared to 91 healthy subjects	HFC% was significantly lower and LFC% significantly higher in patients with depression not on hypnotics compared to patients with depression on hypnotics and healthy subjects
Chien et al. (2013)	Sleep quality of 156 nurses was assessed using the Chinese edition of Pittsburgh sleep quality index	CPC were analyzed and classified into stable vs. unstable sleep. Patients deemed as poor sleepers according to the Chinese edition of Pittsburgh sleep quality index had a significant inverse correlation with the stable sleep ratio as determined by CPC
Lin et al. (2013)	CPC patterns were studied in 13 medical interns and nights when being on call were compared to nights when they were off duty	HFC% significantly decreased during on call nights when sleep deprivation is expected compared to off duty nights
Schramm et al. (2013)	CPC variables were compared between 50 patients with primary insomnia and 36 good sleepers	Primary insomnia patients had lower HFC%, low HFC/LFC ratio, and higher LFC% when compared to good sleepers
Jarrin et al. (2016)	Single arm cohort study of 65 patients with chronic insomnia before and after 6 weeks of cognitive behavioral therapy	Improvement in sleep parameters were associated with lower HFC% contrary to what would be expected. Study is limited by absence of control arm
Thomas et al. (2018)	CPC variables from 128 nights were collected from10 healthy volunteers and compared to 121 nights in 20 patients with insomnia.	Patients with insomnia had increased LFC specifically increased broad-band LFC (LFC_*BB*_)
Hilmisson et al. (2019a)	Prospective cohort of 110 patients with chronic insomnia that have been treated with prescription pharmacological agents for > 3 months and not previously tested for OSA. Home sleep testing showed that 25% had moderate to severe OSA coexistent with their insomnia diagnosis.	Patient with insomnia who were found not to have OSA had less percentage of LFC specifically less LFC_*NB*_ when compared to patients with insomnia who were found to have moderate to severe OSA. There were no significant differences in CPC parameters in patients with insomnia without OSA when compared to patients with insomnia who have mild OSA
Sun et al. (2019)	41 patients with depression studied before and after 2 weeks of antidepressant medications	Increase in HFC was associated with improvement in psychiatric questionnaire scores
Zhang et al. (2021)	CPC variables were compared between 3 groups: 22 insomnia patients with cognitive impairment, 21 insomnia patients with normal cognition, and 15 healthy volunteers	Insomnia patients with cognitive impairment had less HFC and more LFC/HFC ration when compared to insomnia patients with normal cognition and healthy volunteers

### Sleep Apnea Treatment Effects

Successful sleep apnea treatment is expected to increase HFC relative to LFC, including following oral appliance therapy and upper airway surgery (Schramm and Thomas, 2012; Lee et al., 2014, 2016; Choi et al., 2015). A similar pattern is noted in pediatric patients with OSA after adenotonsillectomy (Lee et al., 2012; Chen and He, 2019). The same results are seen with CPAP treatment of OSA (Harrington et al., 2013; Cho and Kim, 2017). Successful treatment of OSA with CPAP is associated with improvement in HFC/LFC ratio (Roche et al., 1999; Shiina et al., 2010). Gilman et al. (2008) randomized patients with heart failure (ejection fraction less than 45%) who had moderate to severe OSA to CPAP vs. usual care. After 1 month the CPAP treated group showed an increase in HFC compared to the control group (Gilman et al., 2008). Harrington et al. (2013) looked at CPAP titration studies and defined successful CPAP titration and optimum CPAP pressures as AHI less ≤ 5 / h of sleep for 30 min during supine REM; higher HFC was found in successful CPAP PSGs and higher LFC in unsuccessful titrations (Harrington et al., 2013).

### Endotyping and Phenotyping Sleep Apnea

Endotypes are the mechanisms which drive pathology, while phenotypes are the expression of these endotypic effects. Multiple driver endotypes are now recognized as important in the pathogenesis of obstructive sleep apnea, including high loop gain, low arousal threshold, airway collapsibility, impaired negative pressure response, and sleep fragmentation resulting in amplified wake-sleep transitional instability (Dutta et al., 2021; Finnsson et al., 2021). Thus, what is considered “obstructive sleep apnea” can be caused by one or more of the above driving mechanisms, which can be classified into anatomical and non-anatomical. High loop gain, reflecting respiratory control instability and an imbalance between input (oxygen and carbon dioxide levels) and output (neural drive to respiratory muscles and upper airway) of the respiratory system, is perhaps the most important non-anatomical endotype. When loop gain is more than 1, self-sustained oscillations are inevitable. The importance of high loop gain is that treatment failure risk is high and options such as oxygen (Edwards et al., 2016; Sands et al., 2018) and acetazolamide (Edwards et al., 2012) can be beneficial. Though mathematical methods can accurately quantify endotypes, analysis of the expressed phenotypes can also accurately identify sleep apnea with high loop gain.

**FIGURE 4:**
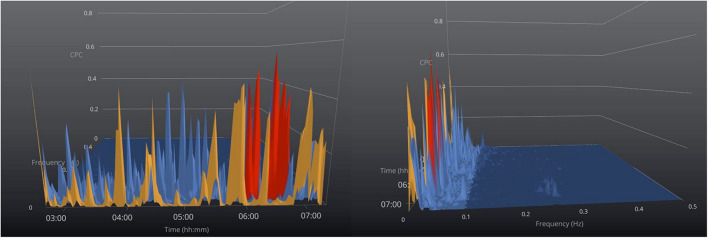
Sleep apnea phenotyping. 3-Dimensional graphical view of the CPC- spectrogram in a patient with severe sleep apnea and no stable (HFC) sleep. Color code: orange = VLFC, blue = e-LFC_*BB*_ (broadband coupling), and red = e-LFC_*NB*_ (narrowband coupling). The offset view (right) shows the narrow dispersion of coupling frequencies induced by periodic breathing toward the end of the recording period, while earlier in the night the e-LFC spectra are “broadly dispersed,” consistent with predominantly obstructive sleep apnea. On the figure to the right the time axis is cut off and the figure is off set to show the narrow band best.

When high loop gain is manifested, the polysomnographic patterns include classic central sleep apnea, periodic breathing, complex apnea with codominant loop gain and airway pathology, treatment-emergent central sleep apnea, and NREM-dominant obstructive sleep apnea. A common theme across all these conditions is self-similar (metronomic timing, identical morphology of consecutive events) of respiratory abnormality (Oppersma et al., 2021), which induce e-LFC_NB_, which is a marker of this expressed high loop gain (Figure 4; Thomas et al., 2007). In a study of 671 subjects with sleep apnea which compared CPC indices to conventional PSG scoring (Thomas et al., 2007), e-LFC_NB_ was associated with respiratory instability during CPAP titration. Since e-LFC_NB_ is a marker of expressed high loop gain and “central” sleep apnea, Ramar et al. (2013) evaluated if could be used as a marker of adaptive servo-ventilation titration success in 106 patients with complex sleep apnea. Overall ASV titration success as defined as AHI < 10/h on ASV was found in 81% of patients and no correlation was found between percentage of LFC_NB_ and ASV titration success (Ramar et al., 2013). One limitation of this study was the use of opiates, which causes ataxic breathing, and is unlikely to cause the exact self-similarity needed to induce e-LFC_NB_. Table 1 (section B) summarizes the studies that used CPC in phenotyping and following response of treatment of sleep apnea.

## Cardiopulmonary Coupling in Other Sleep Disorders

CPC has been used to study other sleep disorders beyond OSA. Patients with insomnia have been shown to exhibit increased LFC even in the absence of sleep disordered breathing (Thomas et al., 2018). It appears that LFC_BB_ is the main LFC pattern seen in pure insomnia so the coexistence of LFC_NB_ should raise the suspicion for coexisting sleep apnea (Hilmisson et al., 2019b). Schramm et al. (2013) studied CPC in a group of primary insomnia and compared to a group of good sleepers. They found increased LFC and a lower HFC/LFC ratio among the insomnia group (Schramm et al., 2013). Zhang et al. (2021) studied insomnia patients with cognitive impairment and found decreased HFC indicating predominance of unstable sleep compared to insomnia patients with normal cognition. However, Jarrin et al. (2016) found that improvements in some sleep parameters in insomnia patients subjected to 6 weeks of cognitive behavior therapy was associated with decreased HFC. One of the limitations of this study was absence of control group. A systematic review of cardiovascular autonomic activity in insomnia patients showed that increased LFC/HFC ratio is a consistent finding in those patients (Nano et al., 2017). Similar findings of increased LFC/HFC ratio were also seen in CPC studies of populations with conditions that would predispose them to secondary insomnia/short sleep durations including: sleep deprivation, fibromyalgia, and periodic limb movement disorder (Sforza et al., 2005; Thomas et al., 2010; Chien et al., 2013; Lin et al., 2013).

Since insomnia is common in patients with uncontrolled psychiatric disorders (Sateia and Nowell, 2004); CPC could be used in studying and tracking treatment response in such patients. In comparison to controls, patients with untreated major depression have reduced HFC and increased LFC (Yang et al., 2011). Sun et al. (2019) studied 41 patients with depression and showed that the increase in HFC following 2 weeks of antidepressant medications treatment was associated with improvement in psychiatric questionnaire scores and suggested that this can be used to predict early response to treatment in such patients. Table 1 (section C) summarizes the studies that used CPC in sleep disorders other than sleep apnea.

## Conclusion

The CPC sleep spectrogram provides a novel window into sleep physiology and key information about sleep during health and disease. Because such data can be obtained from simple/reduced and even contactless signal acquisition methods, it allows studying sleep in greater numbers, and with greater ease, in a wider range of conditions, with nearly limitless repeatability, than typically possible with traditional polysomnograms or current home sleep apnea testing devices.

## Author Contributions

HA and RT wrote the manuscript. All authors contributed to study design and the literature search, and revised the manuscript.

## Conflict of Interest

RT has the following disclosures: (1) Patent for a device to regulate CO_2_ in the positive airway pressure circuit, for treatment of central/complex apnea. (2) Patent and license for an ECG-based method to phenotype sleep quality and sleep apnea (to MyCardio, LLC, through Beth Israel Deaconess Medical Center). (3) Patent, past consultant—DeVilbiss-Drive, CPAP auto-titrating algorithm. (4) GLG Councils and Guidepoint Global– general sleep medicine consulting. The remaining authors declare that the research was conducted in the absence of any commercial or financial relationships that could be construed as a potential conflict of interest.

## Publisher’s Note

All claims expressed in this article are solely those of the authors and do not necessarily represent those of their affiliated organizations, or those of the publisher, the editors and the reviewers. Any product that may be evaluated in this article, or claim that may be made by its manufacturer, is not guaranteed or endorsed by the publisher.
